# Characterization of internal fatigue cracks in aluminum alloys by simulation of phase contrast tomography

**DOI:** 10.1038/s41598-022-09811-8

**Published:** 2022-04-08

**Authors:** Ce Xiao, Jean Michel Létang, Jean-Yves Buffière

**Affiliations:** 1 Laboratoire Matériaux, Ingénierie et Sciences (MATEIS), CNRS UMR5510, INSA-Lyon, 69621 Villeurbanne, France; 2 University of Lyon, INSA-Lyon, Université Lyon 1, UJM-Saint Etienne, CNRS, Inserm, CREATIS UMR 5220, U1294, 69373 Lyon, France

**Keywords:** Metals and alloys, Imaging techniques, Characterization and analytical techniques, Mechanical engineering

## Abstract

Synchrotron Radiation Computed Tomography (SRCT) allows a better detection of fatigue cracks in metals than laboratory CT due to the existence of phase contrast. However the presence in reconstructed images of fringes at the edges of objects generated by Fresnel diffraction makes it difficult to identify and analyze the cracks quantitatively. Simulations of phase contrast synchrotron tomography images containing cracks with different sizes and shapes are obtained by using GATE software. Analyzing the simulation results, firstly, we confirmed that the bright parts with strong contrast in SRCT image are streak artifacts; secondly, we found that the gray scale values within the cracks in SRCT images are related to the crack size; these simulation results are used to analyse SRCT images of internal fatigue cracks in a cast Al alloy, providing a clearer visualisation of damage.

## Introduction

Computed Tomography (CT) is an efficient non-destructive testing technology for observing internal features (cracks, defects, inclusions, etc.) in opaque materials. For the characterization of damage inside metals, two main tomography modes are widely used: attenuation CT and phase contrast CT. With standard industrial sources, the size of the focus spot is usually too large and the X-ray energy spectrum too broad to make it possible to see X-ray interference effects, only attenuation CT is therefore possible. On the other hand, with nanofocus laboratory sources or synchrotron radiation facilities, the X-ray beam is much more coherent, which allows the detector to record more effectively the phase change information of X-rays and phase-contrast is by default observable. For the study of fatigue crack propagation in metals, Fresnel diffraction of X-rays is exploited to enhance the visibility of edges and boundaries within an object^1^. Most common types of phase contrast CT are propagation-, analyser- or grating-based^2^. The opening of fatigue cracks in metals at the crack tip is in the micrometer range, which results in a very low contrast, and Synchrotron Radiation Computed Tomography (SRCT) is therefore crucial to observe sub-voxel features thanks to phase contrast^3^.

However, compared with standard attenuation tomography, for which the gray-scale value of the reconstructed image is proportional to the linear attenuation coefficient of the material, phase contrast tomography brings an increase in image complexity: phase contrast typically generates fringes at the edges of objects in the reconstructed images^3,4^; in addition, streak artifacts are generated^5^. This complexity makes it difficult to precisely identify the cracks in the reconstructed image and quantitatively analyze it^6^. Some reconstruction methods enable phase retrieval in single distance phase contrast tomography: the Paganin method^7^ or the Moosmann method^8^ (named after the 1$^{st}$ author of the corresponding paper). Single-distance propagation-based phase contrast tomography is widely preferred to save time during in-situ experiments^9^. However, for SRCT reconstruction of fatigue cracks in metal, the Paganin method causes blurring of the crack edges; meanwhile, the Moosmann method requires long object-to-detector distances (typically several meters for metals) which are not easily obtained in all beam-lines. Therefore in studies reporting 3D crack images published so far, the classical Filtered Back Projection (FBP) reconstruction method without phase retrival is used as for example in Al alloys^10,11^; in Ti alloys^12,13^; in cast iron^14^; in Mg alloy^15^. The simulated images in this paper are obtained using the FBP method.

**Figure 1:**
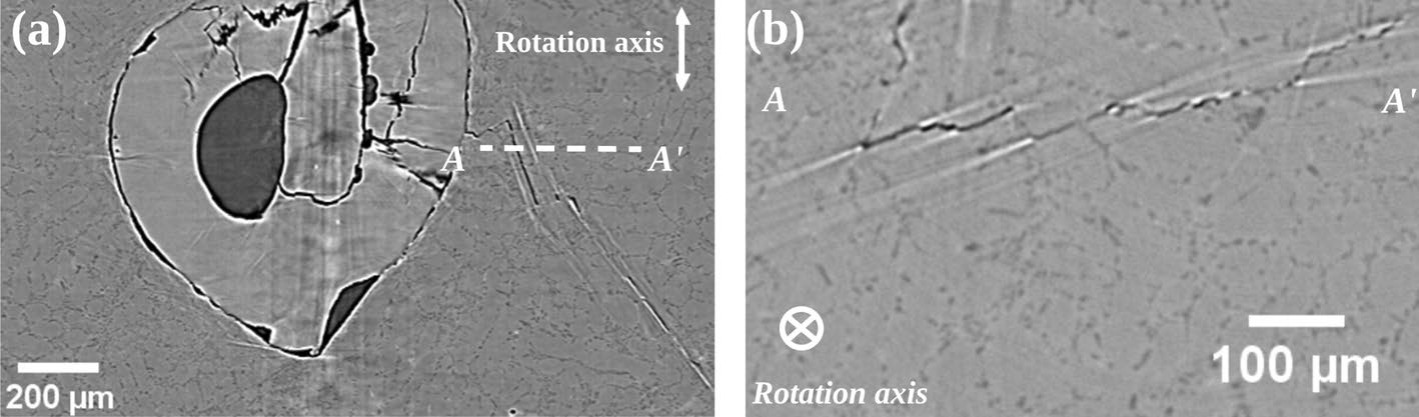
(**a**) Reconstructed image (vertical slice) of a fatigue crack within a cracked aluminum alloy sample obtained at SOLEIL (PSICHE beam-line) with an energy of 29 keV, a distance sample-detector of 15 cm and a voxel size of 1.3 $\upmu$m, the stress direction is parallel to the rotation axis; (**b**) Horizontal slice along the AA$^{\prime }$ dashed white line. In both slices dark lines correspond to voxels belonging to the crack, the brighter parts may be streak artifacts or flat cracks with small voxel opening.

Figure 1 shows a typical reconstructed image of an internal fatigue crack inside a cast aluminum alloy sample. This image has been obtained at SOLEIL (PSICHE beam-line) with an energy of 29 keV, a sample-detector distance of 15 cm and a voxel size of 1.3  $\upmu$m. Because internal fatigue cracks grow without being into contact with ambient air, the crack surfaces correspond to crystallographic slip plane ($\{1 1 1\}$ in that specific case) often inclined with respect to the tensile/sample axis^16,17^. The dark features in this image correspond to voxels with near zero attenuation i.e., belonging to the crack. However, many bright features with strong contrast also appear as white lines in the slices. In vertical slices (the rotation/tensile axis are both vertical), those white lines are parallel to dark lines corresponding to voxels belonging to the cracks (Fig. 1a); in horizontal slices the white lines tend to appear at the end of the dark lines (Fig. 1b). It is difficult to interpret those white features unambiguously. They might be flat internal cracks with small opening, but they may also be streak artifacts. If they correspond to cracks, one clearly needs a criterion to decide when a crack appears dark and when it appears bright. If they are artifacts, one needs to know how they are related to the cracks. For addressing those issues, a simulation approach is needed first.

The simulation of the whole X-ray imaging chain, from the source to the detector can be obtained with different methods which can be divided into probabilistic methods and analytic methods^4^. Some Monte Carlo based methods for modelling the X-ray interactions with the material (e.g. refraction and scattering) have been implemented in Gate^18^ and in Geant-4^19^. In this work, we focused on phase contrast (Fresnel diffraction), therefore, a method proposed by Langer et al.^20^ using a ray casting procedure to obtain integral of the refractive index, as well as an analytical wave optics approach for generating Fresnel diffraction patterns is used to simulate phase contrast tomography. The implementation is in the GATE software (version 8.2 and later).

The purpose of this article is to better analyze the flat crack signatures in SRCT images reconstructed by the FBP method by phase contrast tomography simulations. In a first step, the simulation results allow us to compare a known crack structure (phantom) to the simulated reconstructed image, thereby distinguishing cracks from artifacts. By changing the geometry and size of the crack in the phantom, the relationship between the crack gray value and the crack size was obtained. Finally, cracks observed in experimental SRCT images were reanalysed and a better understanding of the crack path was obtained. To the best of the authors’ knowledge there hasn’t been any systematic study of this type published in the literature yet.

## Results

### Simulation results of flat crack

**Figure 2:**
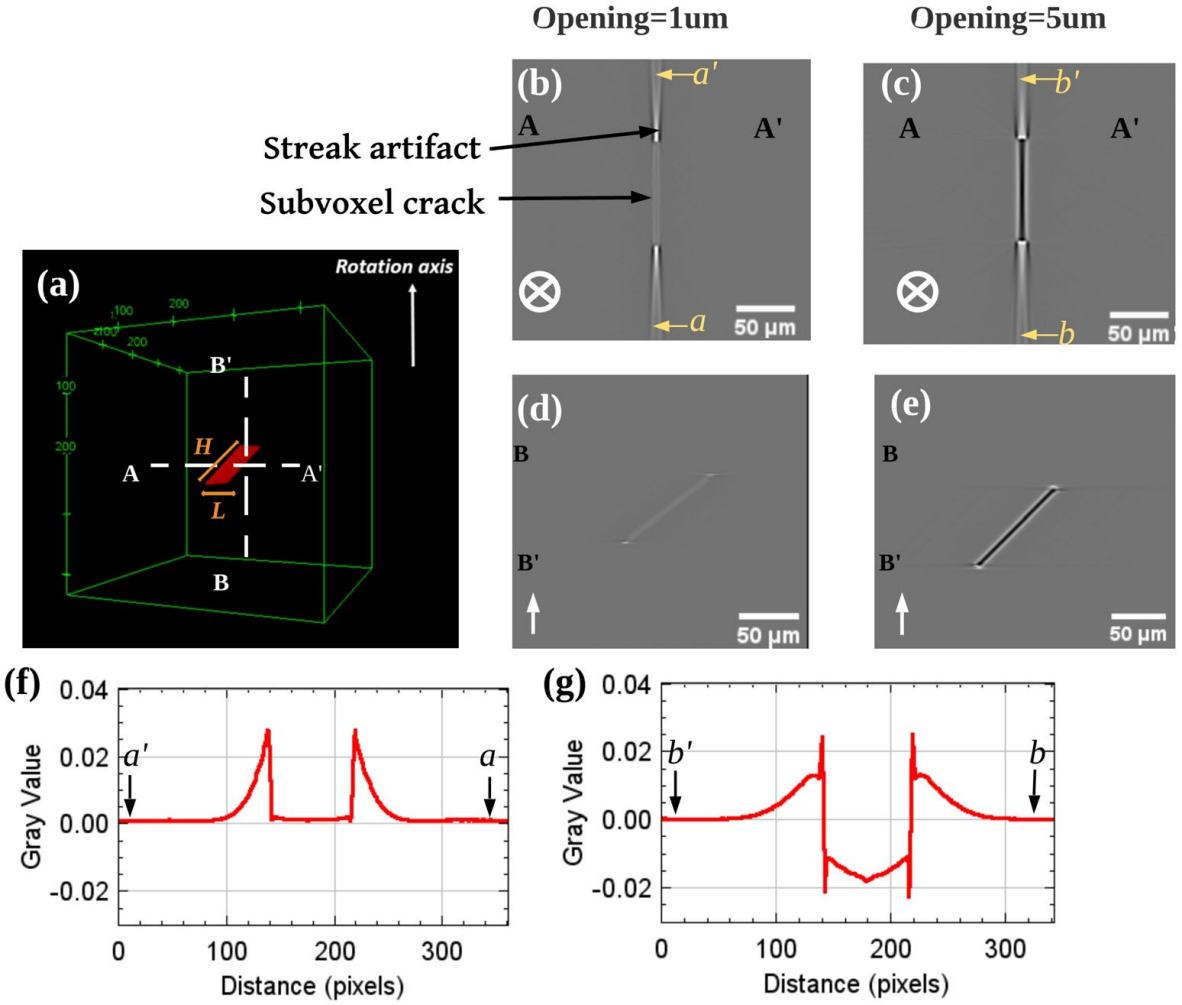
Simulated images obtained with the following imaging conditions: X-ray energy 29 keV, object-detector distance 150 mm, detector pixel size 1.3 $\upmu$m. The crack was assumed to be a plane at $45{^{\circ }}$ to the rotation axis (whose direction is indicated in white in the lower left corner of the images), with a length (L) and height (H) set to 100 $\upmu$m, two crack openings were investigated: 1 $\upmu$m and 5 $\upmu$m, respectively. (**a**) Phantom’s 3D rendering, the white dashed lines AA$^{\prime }$ and BB$^{\prime }$ indicate the position of the horizontal and vertical slices (positioned at the middle of the crack) respectively. (**b**) Simulated horizontal slice (A–A$^{\prime }$) of the crack with sub-voxel opening 1 $\upmu$m; (**c**) Simulated horizontal slice (A–A$^{\prime }$) of the crack with opening 5 $\upmu$m; (**d**) Simulated vertical slice (B–B$^{\prime }$) of the crack with sub-voxel opening 1 $\upmu$m; (**e**) Simulated vertical slice (B–B$^{\prime }$) of the crack with opening 5 $\upmu$m; (**f**) Distribution of grayscale values along the crack (opening 1 $\upmu$m) along the (a–a$^{\prime }$) line in horizontal slices; (**g**) Distribution of grayscale values along the crack (opening 5 $\upmu$m) along the (b–b$^{\prime }$) line in horizontal slices. Bright high-intensity artifacts were observed in both horizontal slices (**b**) and (**c**).

As mentioned above, internal fatigue cracks in the studied cast aluminum alloy propagate on crystallographic planes inclined with respect to the direction of tension^16^. Therefore, in this section our model crack (phantom) is a plane inclined at $45{^{\circ }}$ to the rotation axis (parallel to the load direction), and the sample is a cube ($1\,{\text {mm}} \times 1\,{\text {mm}} \times 1\,{\text {mm}}$) of the AlSi7Mg0.6 as alloy shown in Fig. 2a. The imaging parameters are those used during the in-situ fatigue experiment performed at PSICHE beam-line^10^ X-ray energy 29 keV, object-detector distance 150 mm, detector pixel size 1.3 $\upmu$m.

First, two different crack opening degrees equal to 5 $\upmu$m and 1 $\upmu$m (sub-pixel opening) are studied; the crack length (L) and height (H) are both equal to 100 $\upmu$m. The simulated 3D image of the phantoms is shown in Fig. 2. When the crack opening is 5 $\upmu$m (Fig. 2b), it can be observed that the crack appears dark, and bright streak artifacts appear at both ends of the crack in the horizontal slice with a slightly divergent shape away from the crack. At the end of the crack those artifacts present a very strong intensity which decreases with distance (Fig. 2f). In vertical slices, the white artifacts do not appear at the ends of the cracks (Fig. 2e). When the crack opening is reduced to 1 $\upmu$m (sub-pixel opening), the contrast in the crack is so weak that the crack can barely be distinguished from the matrix by gray-scale values with respect to noise; however the bright artifacts at the ends of the crack in the horizontal slice remain unchanged compared to those at 5 $\upmu$m (Fig. 2b). This seems to indicate that cracks with sub-pixel openings, which cannot be directly observed in horizontal sections, can be revealed by artifacts at both ends, this phenomena will be investigated in the experimental images of the discussion sections. In vertical sections, the cracks remains barely visible and no bright artifacts can be seen at either end (Fig. 2d). The simulation results of an extended flat crack show therefore that the crack’s gray scale values in the reconstructed image are strongly related to its opening, this point is further analysed in the next section.

### Crack gray value in the reconstructed image

**Figure 3:**
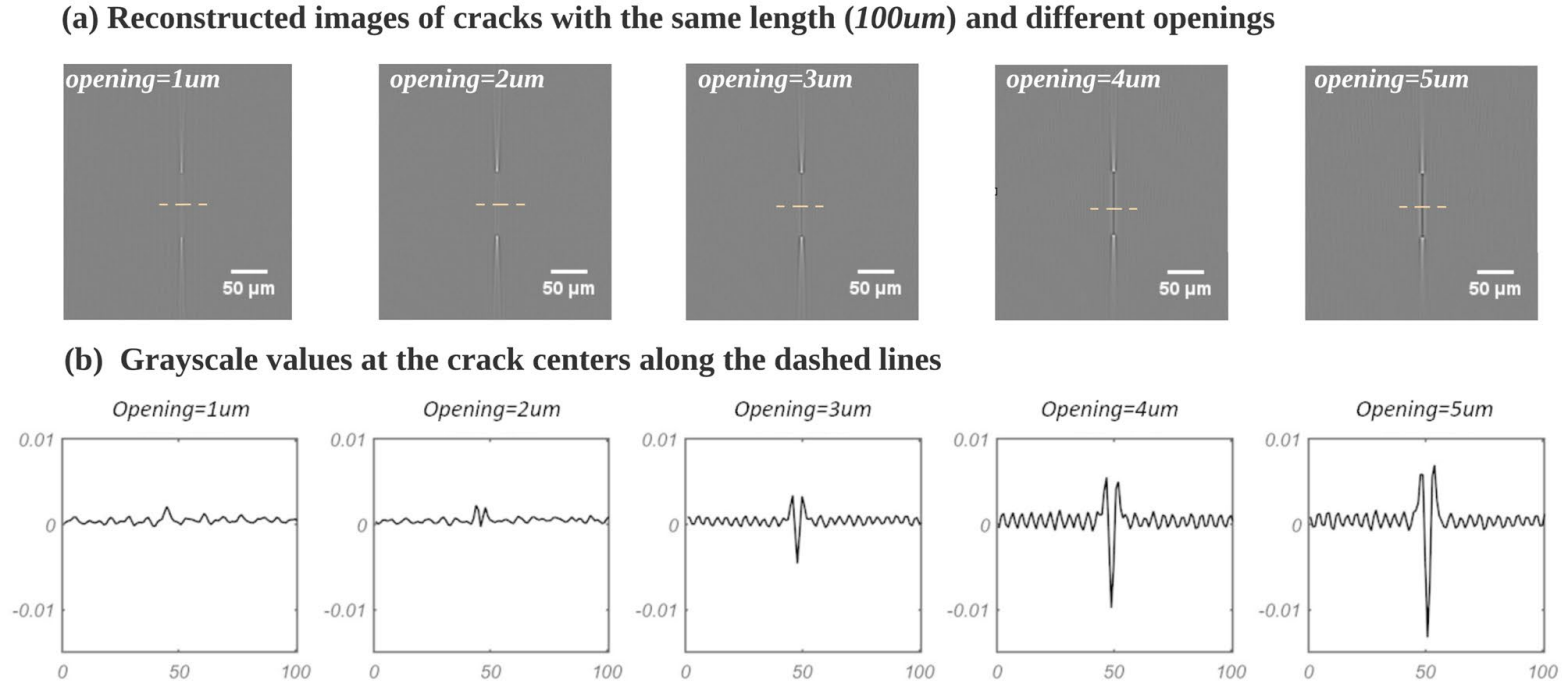
All slices are perpendicular to the axis of rotation: (**a**) Simulated images of cracks with openings ranging from 1 to 5 $\upmu$m; (**b**) Center profile of gray value of each crack: the contrast of the crack (as defined in Eq. 1) in the reconstructed image increases as the crack opening increases.

To further quantify the relationship between the gray value and the opening degree, the crack’s length (L) and crack’s height (H) have been both fixed as 100 $\upmu$m and the opening degree of the crack varies from 1 to 5 $\upmu$m in 1 $\upmu$m steps. The results of these simulations are shown in Fig. 3; the cracks with openings values equal to 1 $\upmu$m and 2 $\upmu$m show a very low contrast in the reconstruction which can hardly be distinguished from the noise. This noise mostly comes from the discretization of the free-space propagation which can experience aliasing artifacts^21,22^. As the degree of opening increases, the center of the crack begins to appear dark, and its contrast increases. Meanwhile, the gray values of the streak artifacts hardly change.

**Figure 4:**
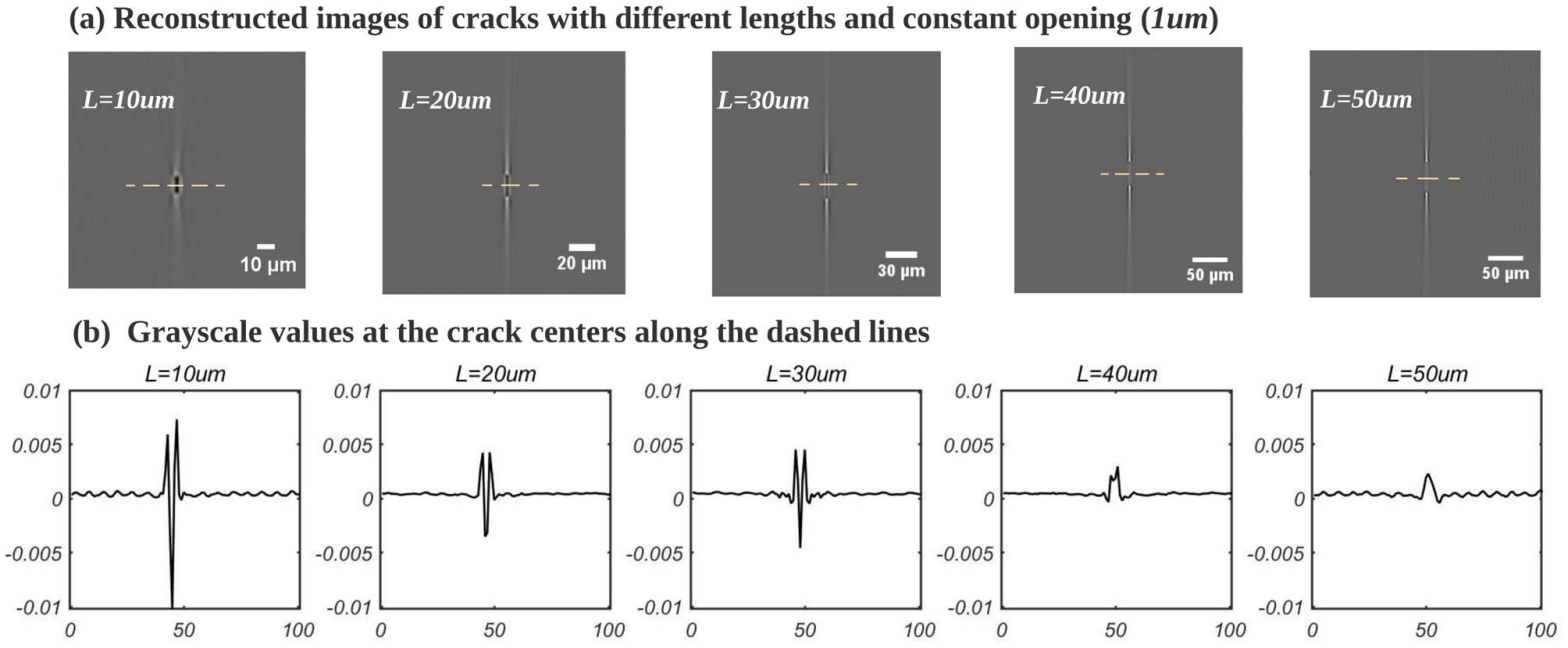
All slices are perpendicular to the axis of rotation: (**a**) The simulated image of cracks with different lengths, 10–50 $\upmu$m; (**b**) Center profile of gray value of each cracks: the contrast of the crack (as defined in Eq. 1) in the reconstructed image decreases as the crack length increases.

Interestingly, it was found that *the gray value in the crack is also related to the crack length*. This is shown in Fig. 4, where the crack openings is fixed to 1 $\upmu$m (sub-pixel), the crack height is unchanged (100 $\upmu$m), and the crack length is gradually changed (10 $\upmu$m/20 $\upmu$m/30 $\upmu$m/40 $\upmu$m/50 $\upmu$m). When the crack’s length is less than 30 $\upmu$m, the crack appears dark with relatively strong contrast. With the increase of the crack’s length, cracks show an increasingly low contrast until they become invisible. The streak artifacts barely change in the meantime. Different values of crack height (100 $\upmu$m/200 $\upmu$m/500 $\upmu$m) were investigated; but in that case the gray value of the crack in the reconstructed image remain unchanged. In summary, the gray values of cracks in the reconstructed image are related to both its lengths and its openings, narrow and long cracks are less likely to be observed in the reconstructed images.

**Figure 5:**
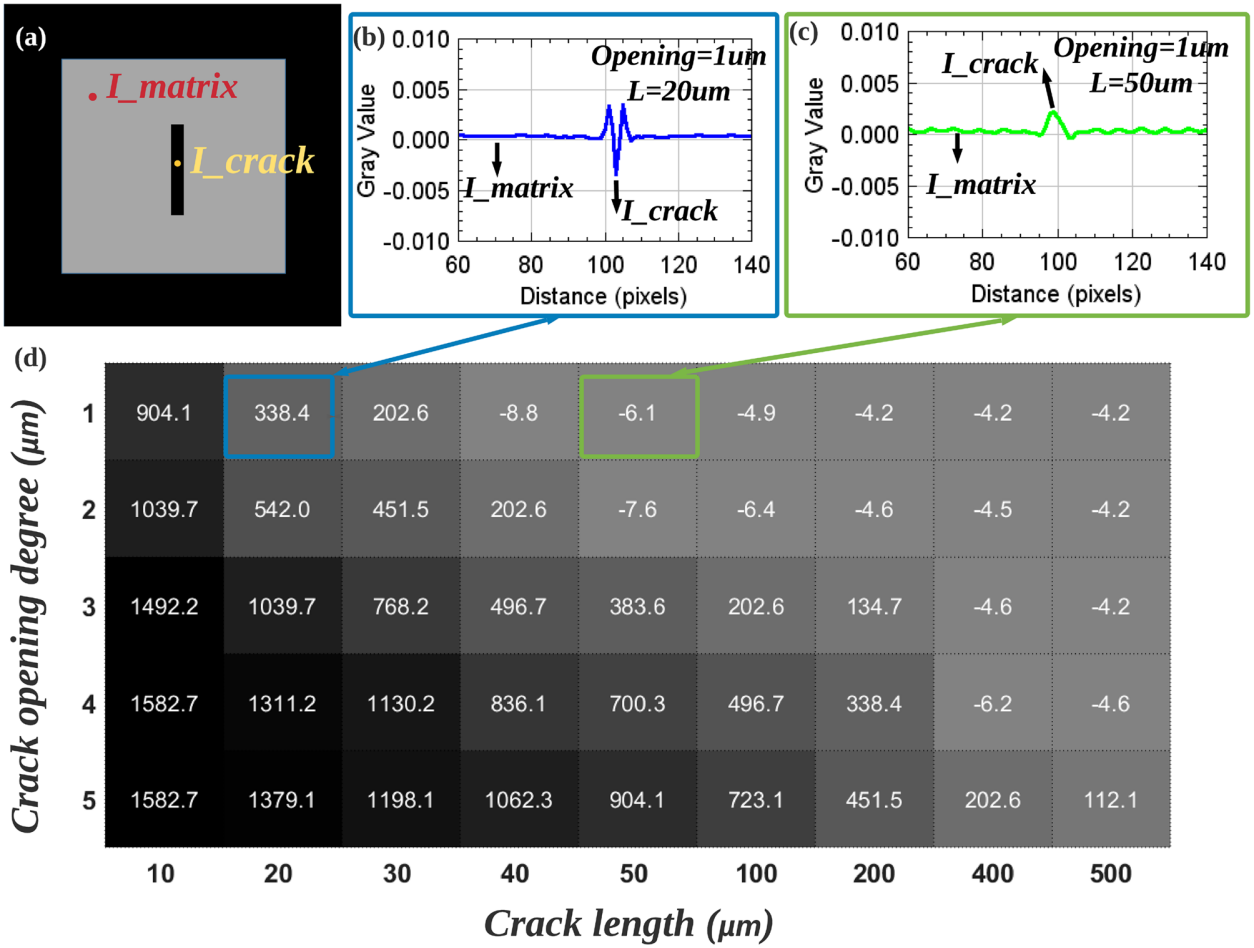
Normalized contrast of cracks with different length/opening in reconstructed images (see Fig. 2 for a definition of the crack geometry). Cracks contrasts are normalized by using the difference between the gray value of the crack center ($I_{crack}$) and that of the gray value of the matrix ($I_{matrix}$), divided by the gray value of the matrix (Eq. 1). (**a**) Horizontal cross-section of scanned specimen, black represents air, gray represents AlSi matrix; (**b**, **c**) Gray value distribution along a profile taken at the center of the crack (opening = 1 $\upmu$m length = 20 $\upmu$m/50 $\upmu$m) in the reconstructed image; (**d**) Table of the normalized contrast, the darker the gray-level in the cell, the easier the cracks are to observe (see the text for details).

Various authors have used 3D tomographic images for characterising the opening (closure) of fatigue cracks under mechanical loading as this parameter has a strong influence on the ability of the crack to propagate during cycling^23,24^. Crack with high levels of closure tend to propagate more slowly during mechanical cycling. Because the gray values in a reconstructed image of a crack are related to both the length and the opening, it is not easy to know the exact value of openings through the gray value. A series of simulation were carried out to quantitatively investigate the effect of the crack size (*L* and opening parameters) on its visibility. Cracks’ contrasts are defined (Eq. 1) as the normalized difference between the intensity at crack center and the intensity of the matrix away from the crack (called $I_{crack}$ and $I_{matrix}$ respectively in Fig. 5a).

1 $$\begin{aligned} \text {Contrast}=\frac{I_\text {matrix}-I_{\text {crack}\_\text {center}}}{I_\text {matrix}} \end{aligned}$$

The results are shown in Fig. 5 (same scan condition as in Fig. 2). A large positive contrast value indicates an intense dark in the center of the crack (Fig. 5b), which corresponds to a large opening and/or relatively short crack length, in which case the crack is easily observed in the reconstructed image. Negative contrast indicates that cracks are not visible and their presence can only be inferred by the streak artifact at both ends (Fig. 5b). A low contrast (positive) indicates a gray level close to that of the matrix at the center of the crack (larger opening and relatively longer length), whereby the center of the crack is not easily detectable, but it should be noted that a high intensity fringe will still be present at the edges of the crack in all cases. A more detailed analysis about the cracks contrast in the reconstructed phase contrast tomography images is given in the “Discussion” section.

### Simulation results of step-like crack

Internal fatigue crack always present a stepped shape^16^ (see for example Fig. 12i). To better understand these stepped cracks in SRCT image, the simulations of two different step-like shapes have been performed (imaging conditions as in Fig. 2).

**Figure 6:**
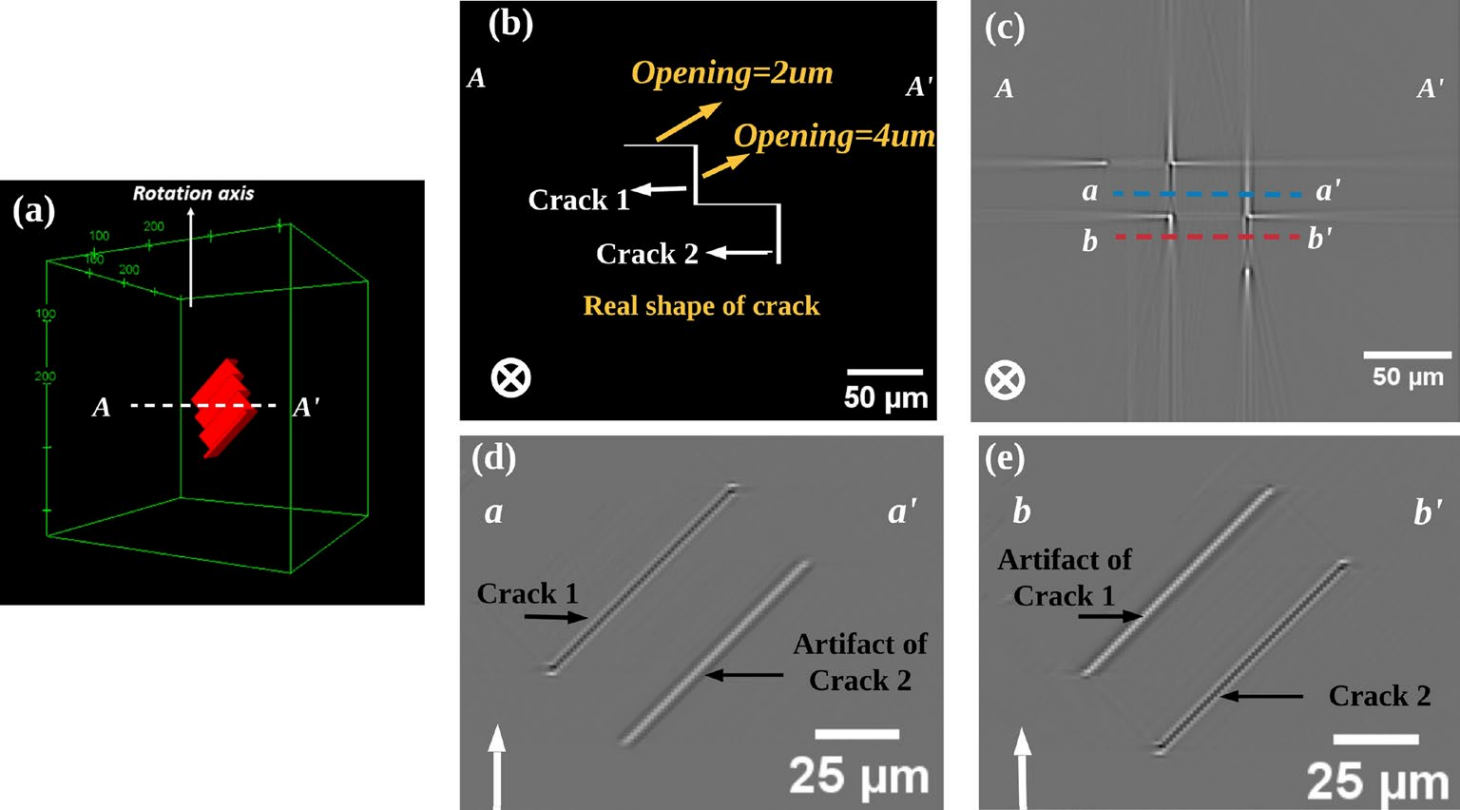
Simulation result (scan conditions as in Fig. 2) of a step-like crack (large steps): (**a**) Phantom’s 3D rendering; 2D section showing the crack in the phantom (**b**) and in the simulated image (**c**) on the plane perpendicular to rotation axis going through A–A$^{\prime }$ line. (**c**) Horizontal slice (A–A$^{\prime }$) of the step-like crack, the blue dashed line (a–a$^{\prime }$) and the red dashed line (b–b$^{\prime }$) mark the positions of the vertical slices shown in (**d**) and (**e**) respectively; (**d**, **e**) Vertical slices of the step-like crack along the dashed blue line and dashed red line shown in (**b**), $\uparrow$ and $\bigotimes$ indicate the rotation axis.

The first simulated results are shown as Fig. 6. In this case, the cracks are assumed to be in step-like shape at $45{^{\circ }}$ to the rotation axis, the step length of the crack is 50 $\upmu$m (relatively large compared to crack size), and the crack opening is set to 2 $\upmu$m or 4 $\upmu$m (not uniformly, as shown in Fig. 6b). In the reconstructed horizontal slice (Fig. 6c), crack segments with an opening of 2 $\upmu$m appear with low contrast and are barely visible, while bright artifacts appear at each end of these segments. Crack segments with a 4 $\upmu$m opening, appear dark, again with bright streak artifacts at both ends. Noteworthy, the artifacts have the similar step shape as the cracks, but they are offset from the location of the cracks, which may cause misleading analysis of the experimental crack images. In the vertical re-slice (orthogonal slice through the defined path within the volume) at the blue dashed line (Fig. 6d), a dark and a bright segment can be observed: the black part corresponds to the crack (marked as crack 1), while the bright part corresponds the artifact generated by crack 2. Equally, the artifacts generated by crack 1 can be found in vertical slices (re-slice at the red dashed line, see Fig. 6e). This indicates that the white artifact in the vertical slice comes from the artifact appearing at the end of the crack on the horizontal slice, which represents the presence of a crack in the neighboring slices.

**Figure 7:**
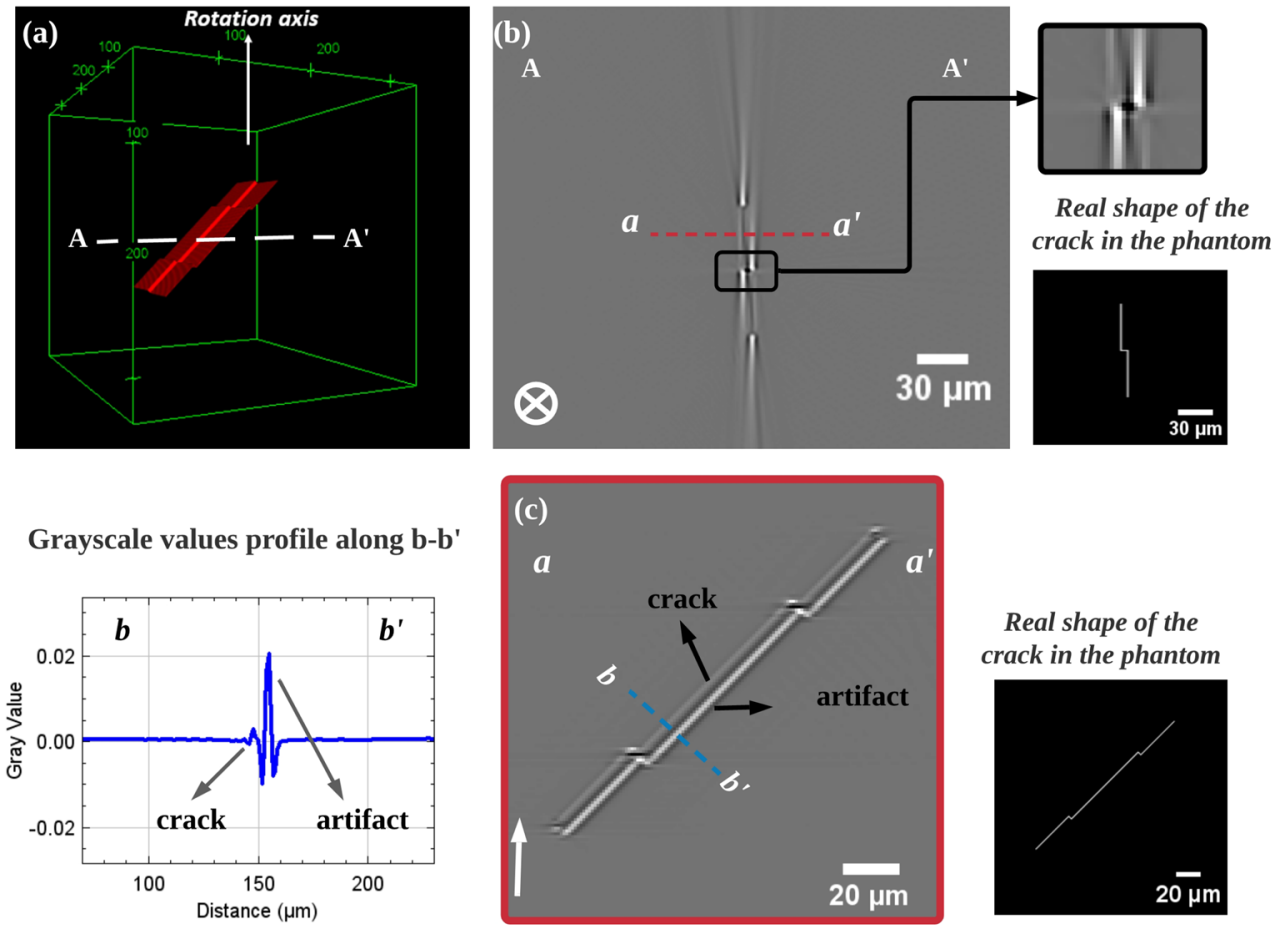
Simulation result of step-like crack (the step size is smaller than in the case of Fig. 6), scan conditions is the same as in Fig. 2: (**a**) Phantom’s 3D rendering, the white dashed line AA$^{\prime }$ indicate the position of the horizontal slice; (**b**) Horizontal slice (A–A$^{\prime }$) of the step-like crack, the red dashed line (a–a$^{\prime }$) mark the positions of the vertical slices (**c**), and the shape corresponding to the cracks is shown next to this image; (**c**) Vertical slices of the step-like crack along dashed red line in (**b**), the shape corresponding to the cracks is shown next to this image and the distribution of gray-scale values along the dashed line is displayed on the left side of the current image, $\uparrow$ and $\bigotimes$ indicate the rotation axis.

Another simulation with smaller steps (5 $\upmu$m) of the step-like crack has been carried out, and the results are shown in Fig. 7. In the horizontal slice (A–A$^{\prime }$), crack segments have a length of 50 $\upmu$m and a opening of 2 $\upmu$m and they appear with low contrast, while bright streak artifacts still appear at the end of the crack (Fig. 7b). Interestingly, cracks appear dark when they change direction, which is consistent with the previous observations that the contrast of the crack increases with decreasing length. In the vertical slice along the red dashed line (a–a$^{\prime }$) (Fig. 7c), cracks and artifacts appear very close in location and have the same shape; the difference is that the contrast of the artifacts is much greater than that of the cracks. This indicates that in vertical slices all bright parts with a strong contrast are artifacts.

## Discussion

### Source of streak artifact

Streak artifacts were observed in several studies using SRCT but their exact origin was not clearly identified. Streak artifacts created by the presence of a metallic implant in phase contrast tomography images of the human brain are discussed for example by Croton et al.^5^. Those authors suggest that the main source of the artifacts may be the imperfect response of the detector. For our simulation results, however the streak artifact at the end of the crack is observed *under ideal detector conditions*, which indicates that its origin might be different from that suggested by Crotron et al. Similar streak artifacts were also observed by Madonna et al. in phase contrast tomography images of rock^25^. Those authors consider that the source of the artifacts is the exponential edge-gradient effects (EEGE) in FBP reconstruction process. Joseph et al. suggest that the streak artifact is created by non-linear error between the recorded projection and the linear spatial averaging of integral of attenuation coefficient^26^. Furthermore, the magnitude of this non-linear error is related to how fast the projection changes on the detector. Thus streak artifacts are more likely to appear at the edges of sharp, high-contrast heterogeneity.

To further investigate the source of the streak artifacts, we simulate phase-contrast tomography with the Weak absorption Transport of Intensity Equation model (WTIE) (Eq. 2). This relatively simple analytical model has been used in the literature to simulate the propagation of X-rays in free space^27^. In contrast to the Fresnel Propagator’s model (Eq. 10), the WTIE model requires the following conditions to be respected: near-field diffraction and slow attenuation variation compared to the phase.

For a monochromatic beam of wavelength, in WTIE model the diffracted intensity writes:

2 $$\begin{aligned} I_{D}({\varvec{x}})=I_{0}({\varvec{x}}) \exp \left( -\frac{\lambda D}{2 \pi } \nabla ^{2} \varphi ({\varvec{x}})\right) \end{aligned}$$

$I_{D}$ the intensity recorded by the detector after a propagation distance *D*, $I_{0}$ the corresponding intensity without any propagation, $\nabla ^{2} \varphi ({\varvec{x}})$ the Laplacian of the phase at pixel ${\varvec{x}}$.

**Figure 8:**
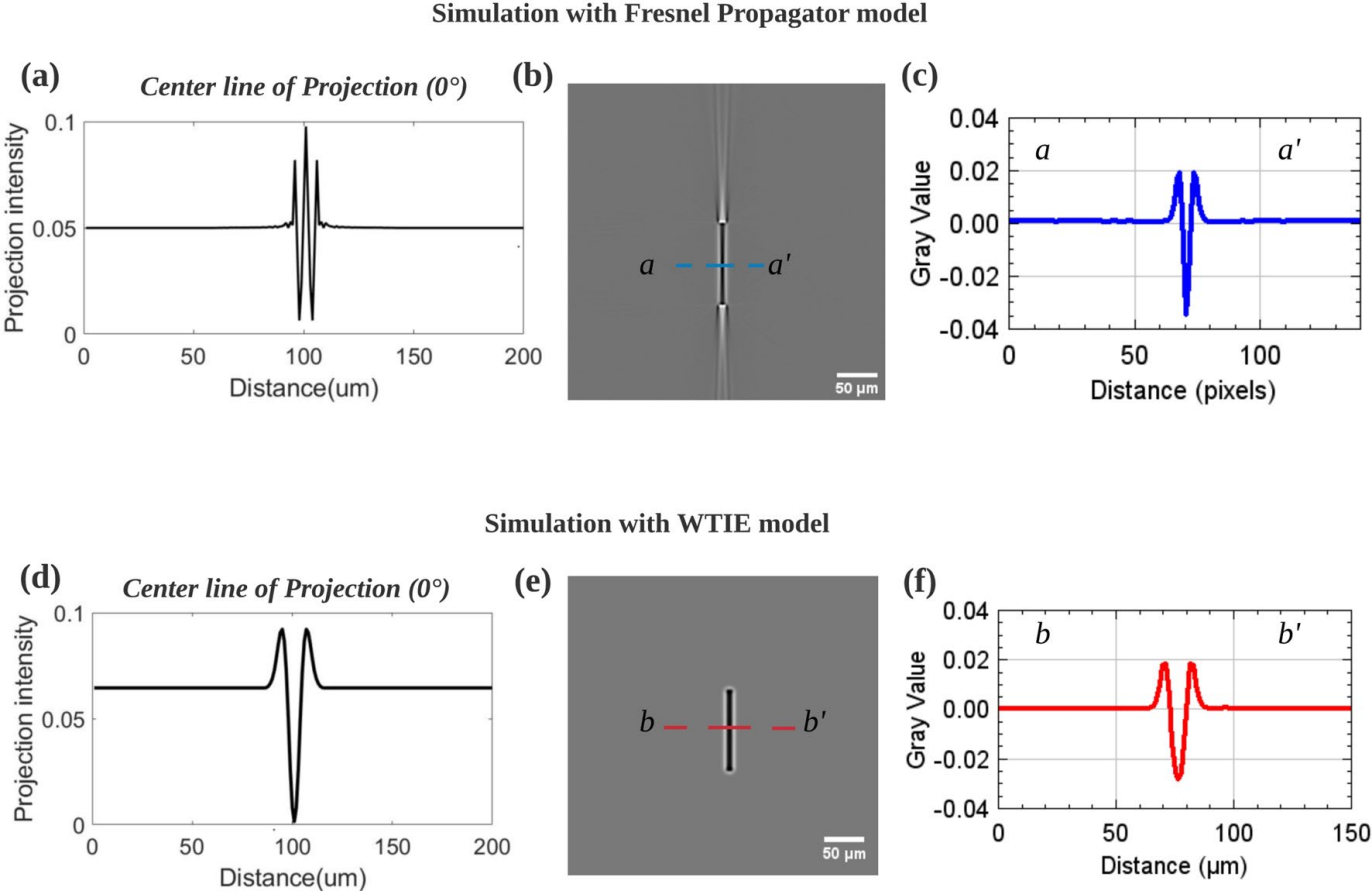
Comparison of simulation results of two models for phase contrast formation with the same phantom and scan conditions: (**a**) Profile of center line of the first projection (0$^\circ$ for which X-ray is incident along the length of the crack) simulated by the Fresnel Propagator method; (**b**) Reconstructed image of a flat crack simulated by the Fresnel propagator method (same phantom as in Fig. 2); (**c**) Distribution of grayscale values along the dashed blue line (a–a$^{\prime }$) in (**b**); (**d**) Profile of center line of the first projection simulated by WTIE model; (**e**) Reconstructed image of a flat crack simulated by WTIE model (same phantom and scan conditions as (**b**)); (**f**) Distribution of grayscale values along the dashed red line (b–b$^{\prime }$) in (**e**).

The simulation results of the Fresnel propagator (implemented in GATE software) and that of the WTIE model with the same phantom and scan conditions are shown and compared in Fig. 8. Streak artifacts are observed in the reconstructed images simulated using the Fresnel Propagator (Fig. 8b), while they are not present in the reconstructed images simulated using the WTIE model (Fig. 8e). The observation of their first projection (0$^\circ$, X-ray parallel to the crack length direction) reveals that the cracks generates more waves in the projection obtained with the Fresnel propagator simulation (in this section a flat field correction is applied to the projections and the log of the image is displayed), which is consistent with 1D Fresnel diffraction patterns^28^; in contrast, in the projection obtained with the WTIE model simulation, because of the simplified linearized formulation, only one simple wave trough is observed. Besides, it is worth noting that the distribution of gray values of cracks in the reconstructed image is almost the same for both models (Fig. 8c,f).

**Figure 9:**
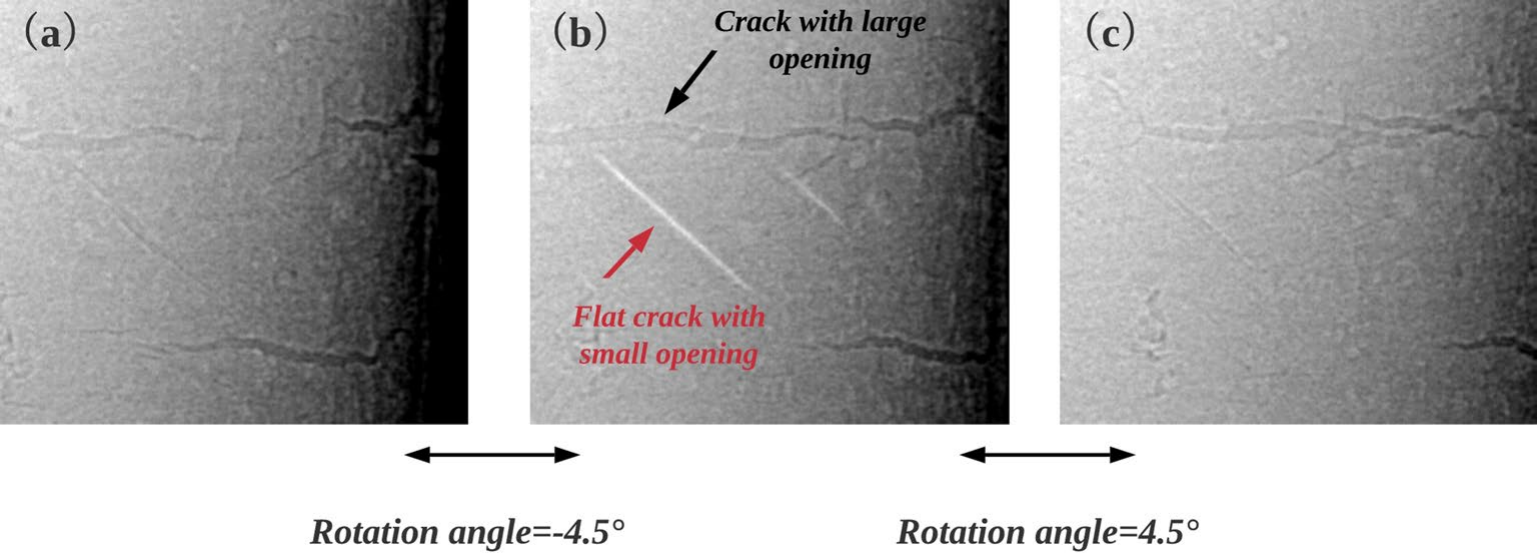
Experimental projections of one SRCT experiment (performed at ESRF and under same imaging conditions as in Fig. 1). (**a**–**c**): Three projections 4.5$^\circ$ apart. Internal flat cracks with small opening appear white on a limited number of projections (less than 10$^\circ$ range); Surface cracks with larger opening appear black with slight white fringes at their edges.

As shown in Fig. 8a, when the Fresnel Propagator is used to generate the projections of the model crack, a white (bright contrast) peak can be observed at the crack center on the projection along the crack length direction (called orthogonal projection) and also in a few projections before and after this rotation position (about $10{^{\circ }}$ angular range). During the back projection stage of the FBP reconstruction, a white thin line (high attenuation) appears which eventually leads to the streak artifact at the crack extremities. On the contrary, when the WTIE model is used to generate the projections of the model crack a black peak (dark contrast) appears at crack center in a few orthogonal projections. Experimental projections of a flat crack are shown in Fig. 9. The images have been obtained at ESRF and under same scan conditions as Fig. 1. It can be seen on Fig. 9 that the flat cracks appear white in the projection during a limited angular range, which is consistent with the results of the Fresnel propagator model simulation but not with those of the WTIE model. The almost perfect impulse response of the detector used in the simulation fits quite well the response of the experimental detector (see for example^29^ which describes a single pixel PSF detector used at ESRF). The WTIE model, in which the nonlinear Fresnel propagation (FP) process is simplified by linearization of the transmittance function via Taylor expansion^30^, generates a smoother signal on the detector. The key point is that this model predicts a dark signal in the interior of the crack (because it uses the Laplacian operator of the phase) while experimentally we observe (Fig. 9b) a white signal. Therefore, when the impulse response of a detector is close to ideal, the FP model gives a more realistic estimate of the crack signature than the WTIE model. It should also be noted that the very flat and thin cracks observed on Fig. 9 are a special case of fatigue cracks: they are extremely flat because they form on crystallographic planes and grow under vacuum. In the general case, however, fatigue cracks tend to initiate *at* and grow *from* the sample surface. Such cracks are not very flat. Also, under load, for a given size, surface cracks have a larger opening that internal ones. As a results, surface cracks appear black in all projections with a slight white fringe at their edges (as shown in Fig. 9 and indicated by a black arrow). These cracks appear black in the reconstructed images, with white fringe at their edges and no streak artifacts.

### Gray value of cracks in reconstructed images

In the above results section, it was observed that *the gray value of the crack in the reconstructed image obtained by phase contrast tomography is related to the crack’s opening and length*. In the above subsection, it has been found that, even if the presence of the white streaks at the end of the cracks is not reproduced by the WTIE model (Eq. 2), this model is however able to reproduce fairly accurately gray-scale values at the center of the crack. The pixels gray values in the reconstructed SRCT image given by the WTIE model write:

3 $$\begin{aligned} g(x, y, z)=\mu (x, y, z)-D\left( \frac{\partial ^{2}}{\partial x^{2}}+\frac{\partial ^{2}}{\partial y^{2}}+\frac{\partial ^{2}}{\partial z^{2}}\right) \delta (x, y, z) \end{aligned}$$

where the first term $\mu$ corresponds to the linear attenuation coefficient at voxel (*x*, *y*, *z*), and the second term is related to the second derivative of the refractive index decrement $\delta (x, y, z)$.

**Figure 10:**
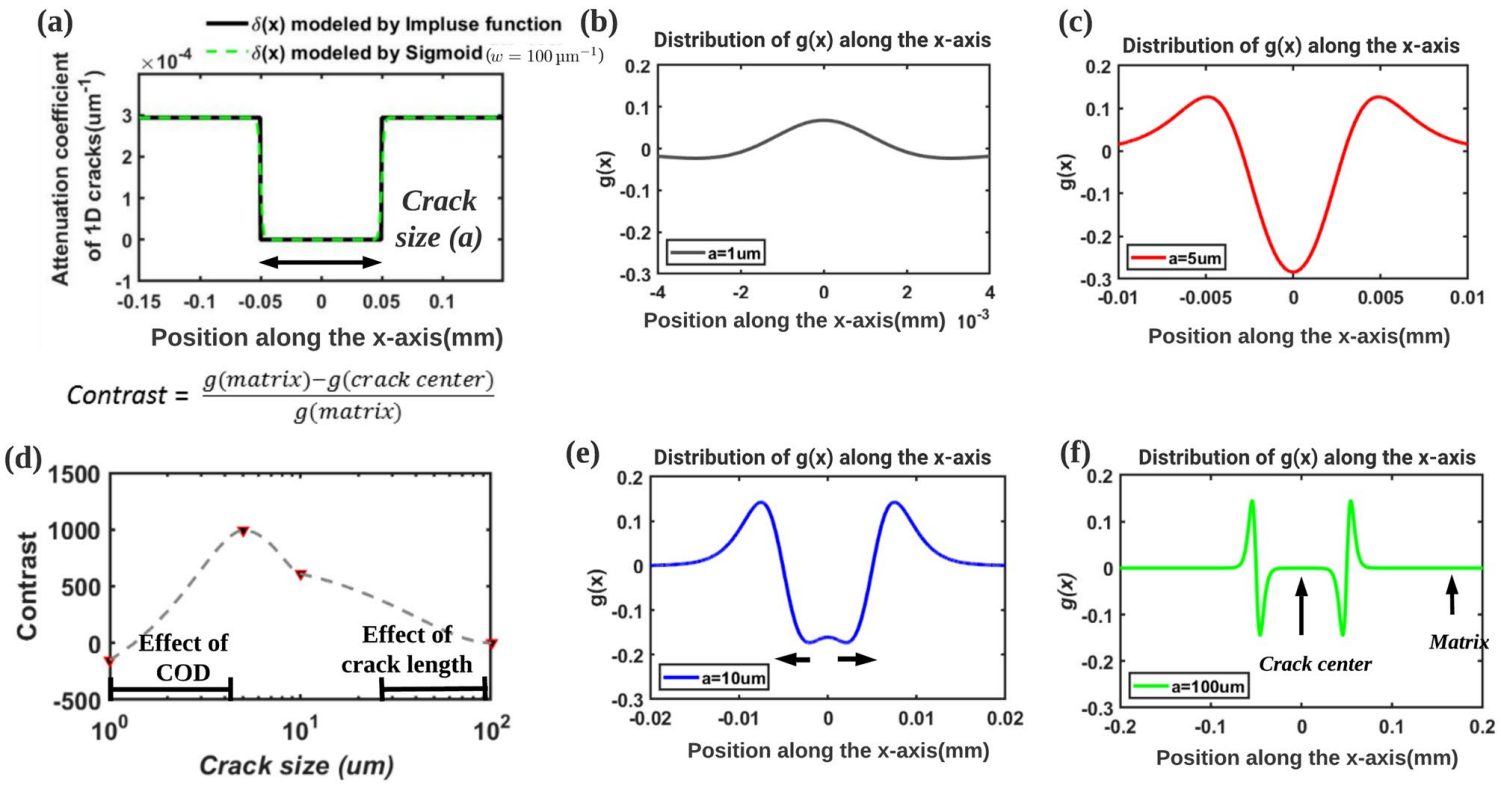
The simplified one-dimensional solution of WTIF model (Eq. 4) is used to discuss the relationship between the crack gray-scale values and the crack size. (**a**) Attenuation coefficient of a one-dimensional crack along the X-axis: the black line is the ideal case (modeled by impulse function), the dashed green line is the simplified case (modeled by a Sigmoid function with $w={100}\,{\upmu }m^{-1}$; (**b**) Distribution of g(x) along the x-axis (crack size = 1 $\upmu$m); (**c**) Distribution of g(x) along the x-axis (crack size = 5 $\upmu$m); (**d**) Normalized contrast (Eq. 1) of crack center for different crack sizes; (**e**) Distribution of g(x) along the x-axis (crack size=10 $\upmu$m); (**f**) Distribution of g(x) along the x-axis (crack size=100 $\upmu$m).

Assuming that the crack is planar and lies in a *yz* plane, its profile of attenuation coefficient along the *x*-axis (X-ray direction) is shown in Fig. 10a. Therefore, the above equation can be simplified to one dimension (Eq. 4),

4 $$\begin{aligned} g(x)=\mu (x)-D\left( \frac{\partial ^{2}}{\partial x^{2}}\right) \delta (x) \end{aligned}$$

and the refractive index decrement $\delta$ can be expressed using a unit step function ${\text {U}}(x)$ (Eq. 5):

5 $$\begin{aligned} \delta (x)=\delta _{\text {Al}}\left( 1-({\text {U}}(x+0.5 a)-{\text {U}}(x-0.5 a)\right) \end{aligned}$$

where *a* is the crack size in the *x* direction.

In order to avoid discontinuity problem in calculating the derivative, a sigmoid function is used instead of the unit step function (Eq. 6), where *w* in the sigmoid function represents the slope; the larger the value of *w*, the closer to the step function *U*. The simplified curve reproduces Eq. (5) when $w={100}\,{\upmu }m^{-1}$ (see green curve in Fig. 10a) and therefore:

6 $$\begin{aligned} {\text {U}}(x) \approx \frac{1}{1+e^{-w x}} \quad \text{ when } w \text{ is } \text{ large } \end{aligned}$$

The second derivative is calculated using Eq. (6), and the expression of the gray value in the reconstructed image reads:

7 $$\begin{aligned} g(x)=\mu (x)-D {\delta _{\text {Al}}}\left( \frac{w^{2}\left( e^{-w\left( x-\frac{a}{2}\right) }-1\right) e^{-w\left( x-\frac{a}{2}\right) }}{\left( 1+e^{-w\left( x-\frac{a}{2}\right) }\right) ^{3}}- \frac{w^{2}\left( e^{-w\left( x+\frac{a}{2}\right) }-1\right) e^{-w\left( x+\frac{a}{2}\right) }}{\left( 1+e^{-w\left( x+\frac{a}{2}\right) }\right) ^{3}}\right) \end{aligned}$$

here $\delta _{\text {Al}}$ is the refractive index decrement of aluminum at 29 keV, $\mu (x)$ is the attenuation coefficient, its value in the air is taken equal to 0. Supposing $w={100}\,{\upmu }m^{-1}$, we then draw the profile of *g*(*x*) when the crack size *a* takes different values.

The result is shown in Fig. 10: when the crack size is small compared to the detector pixel size (e.g. $a={1}\,{\upmu }m$), the scanned object can be almost regarded as a homogeneous object with very small values of second derivative of the refractive index. Therefore, the gray value at the crack center in the reconstructed image is close to that of the matrix (Fig. 10b). This explains why the cracks are invisible when the crack opening is sub-pixel. When the crack size increases to several times of the pixel of the detector (e.g. $a={5}\,{\upmu }m$), the second derivative of the step function at both edges (called sharp signals in the following) of the crack seems to have an effect on the center of the crack (Fig. 10c), and the closer to the center, the more significant the contrast. At this stage, the larger the crack’s opening, the larger the contrast of crack in the reconstructed image (Fig. 10b,c). As the crack size continues to increase (e.g. $a={10}\,{\upmu }m$), it can be observed that the sharp signals generated by the crack edge begin to separate, whereby the contrast in the center of the crack begins to decrease (Fig. 10e). When the crack size continues to increase to several hundred times of the detector pixel (e.g. $a={100}\,{\upmu }m$, when the distance between the two crack surface becomes large, it should be called crack size instead of crack opening), the sharp signals are far enough from the crack center to produce a contrast at crack center close to the attenuation coefficient of air (ie about 0). In this case, the normalized contrast (Eq. 1) in Fig. 5 will be 1. However, the sharp signals appear at the two edges of the crack can help to observe the crack edge (Fig. 10f). This may explain that the gray value of cracks in the reconstructed image decreases with the increase of crack length. In a nutshell as shown in Fig. 10d , the contrast at the crack center increases with increasing size and decreases when the size of the crack is in the range of several tens of pixels.

**Figure 11:**
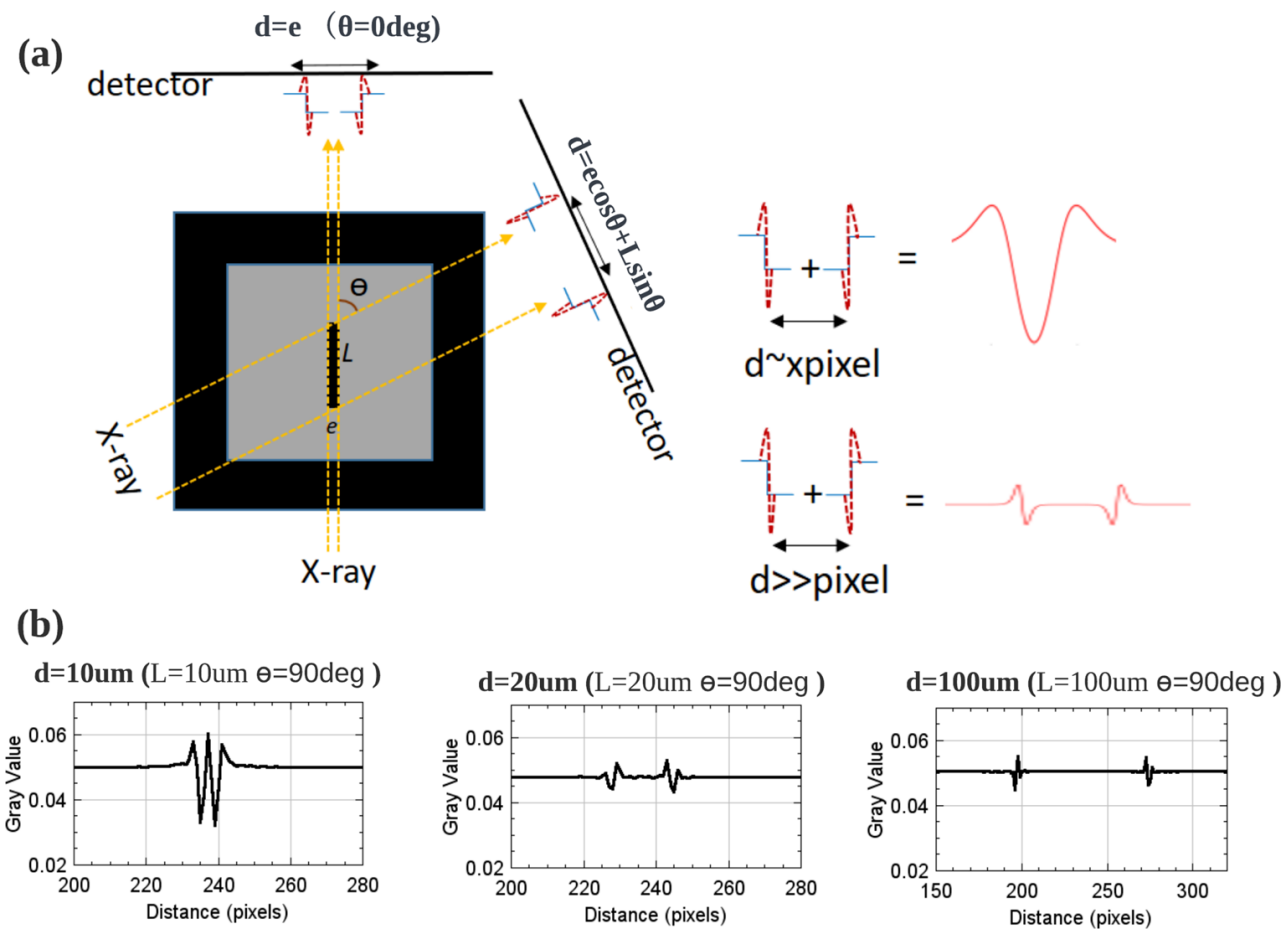
Qualitative explanation of the effect of crack length on the gray-scale values of the cracks in the reconstructed images. (**a**) Schematic diagram of a crack acquisition during scan. In phase contrast tomography, the edges of the crack (marked in blue) generate sharp signals on the detector (marked in red), and an interaction when they are close, causing a high intensity on the detector (at the center of the crack); (**b**) Simulated intensity signals generated at the detector for a given angle ($\theta ={90}^\circ$) and different lengths of cracks (L = 10 $\upmu$m/20 $\upmu$m/100 $\upmu$m, e = 3 $\upmu$m).

The signal generated by the edges of the crack on the detector during the acquisition of the projections can intuitively explain the surprising decrease of the crack gray-scale value with increasing crack length shown previously. In phase contrast tomography, interference patterns related to Fresnel diffraction appears on the detector (called sharp signal and marked using red dashed line in Fig. 11a) at the edge of the object^27^. As explained above, in case of crack phase contrast imaging, the intensity on the detector varies with the distance between the sharp signals generated at the two edges of the crack. During the rotation, the distance between the sharp signals for the projection at angle $\theta$ is shown in Eq. (8):

8 $$\begin{aligned} d= e \cos \theta + L \sin \theta \end{aligned}$$

where *e* denotes the crack opening, *L* denotes the crack length and $\theta$ denotes the angle between X-ray and crack length direction (Fig. 11). When two sharp signals are in close proximity to each other (at a distance *d* of about a few pixels), interference between the sharp signals occurs which render the crack in the projection with a high dark contrast. The smaller the crack length *L*, the greater will be the proportion of projections (ie a larger range of $\theta$ values) for which interference between the ends of the crack will occur. In contrast, when the sharp signals are far from each other, no interference between the sharp signals takes place and the detectors record only the attenuation of the matrix which causes a decrease in the gray value of the cracks in the reconstructed image. For the internal fatigue cracks (flat crack) we are discussing, the crack length is much larger than the crack opening: when the crack length increases, fewer projections will show interferences between the crack edges and the gray value of the crack in the reconstructed image will tend towards that of the matrix. As shown in Fig. 11b, the projections recorded by the detector (simulated by the Fresnel propagator model) for different crack lengths (L=10 $\upmu$m/20 $\upmu$m/100 $\upmu$m) demonstrate the decrease in the intensity of the crack center as the length increases.

### New analysis of internal cracks in experimental images

**Figure 12:**
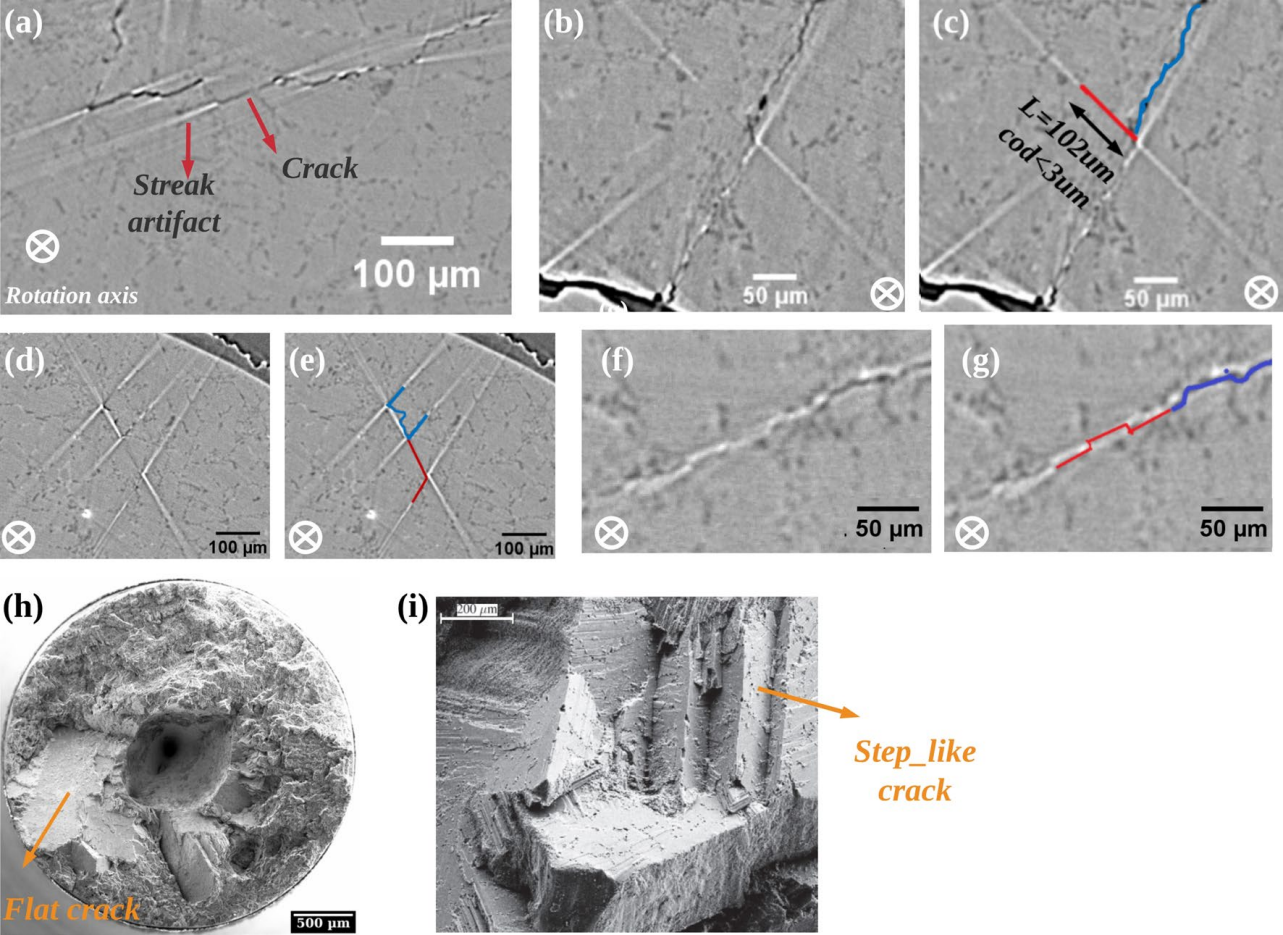
Analysis of cracks and artifacts in horizontal slices of experimental scans: the experimental SRCT images of a cracked AlSi7 specimen which have been obtained at SOLEIL PSICHE beamline (**a**–**e**) and SLS TOMCAT beamline (**f**, **g**) show the same streak artifacts as in the simulated image. The scans conditions of (**a**–**e**) are completely identical as in Fig. 2; the scans conditions of (**f**, **g**): energy 30 keV, distance between the sample and the detector 15 cm and voxel size 1.6 $\upmu$m. All slices are horizontal slices (perpendicular to the rotation axis). (**a**) The same 2D experimental horizontal slice as in Fig. 1b, flat crack with dark and white streak artifact at the ends of the crack can be observed. Tomography images of various parts of the crack are shown in (**b**, **d**, **f**). On these images a red line has been added to indicate (based on the simulation results) that a crack is present in the sample (**c**), (**e**, **g**) and blue lines have been added to the crack segments which can be unambiguously determined (black voxels). (**h**, **i**) SEM observation of the fracture surfaces of the fatigue tested specimen at two different magnifications.

In the previous sections, flat cracks as well as stepped cracks have been simulated. By observing the specimen fracture surface using SEM (Fig. 12h), it was found that the crack surface is extremely flat. They propagate from an internal defect until the specimen fracture. With the help of the simulation results, the experimental images (obtained at SOLEIL PSICHE beamline and SLS TOMCAT beamline, detailed scan conditions can be found in method section) can be better interpreted. First we analyze the cracks and artifacts in horizontal slices (i.e. perpendicular to the rotation axis): as shown in Fig. 12a (same image as in the introduction section), based on the simulation results it is determined that the dark parts of the image are cracks, and the bright parts with strong contrast (located at the ends of the cracks) are artifacts. It can be seen that the gray-scale value of the crack segment is variable, for example the crack marked with a red arrow has a lower contrast than the neighboring crack segments. This lower contrast could be interpreted as a locally smaller opening, but the simulation results show that the decrease in contrast may also equally be due to the local planar shape of the crack on a relatively long distance. This clearly shows the difficulty in trying to assess local crack closure levels from tomography specially on such flat cracks. When the size of the flat crack increases, the simulation result shows that the crack almost disappears in the reconstructed image. This is observed in Fig. 12b, where one can nevertheless detect the crack presence from the streak artifacts which appear at both ends (as in Fig. 12c). The length of this crack segment can be obtained by measuring the distance of the artifacts on both sides (102 $\upmu$m); based on Fig. 5, a maximum opening of the crack of 3 $\upmu$m can be inferred.

Equally, step-like cracks with different sizes can be found in the experimental images. As shown in Fig. 12d and e (obtained at SLS), the above simulation results (Fig. 6) help us to explain that the particularly bright parts in horizontal slices are all artifacts although they present a step shape similar to that of the crack and the actual cracks segments appear between every two streak artifacts. Similar step-like cracks were also found in the SEM observation of the fracture surfaces of the tested specimens (Fig. 12i). Figure 12f is a horizontal slice obtained at another synchrotron X-ray source (SLS) for another AlSi7 specimen aalso containing an internal fatigue crack, the scanning conditions are almost similar to those of the simulation. Small-scale step-like cracks are present in this reconstructed horizontal slice: before obtaining the simulation results (Fig. 7), all bright segments in this slice were considered as cracks; nevertheless, the invisible flat cracks (marked as red segments in Fig. 12g) are now positioned in between the series of streak artifacts, with a certain offset; eventually the crack size is smaller than what was first assumed. In summary, with the help of the simulation results, the fatigue cracks can be more accurately identified in horizontal slices and ***even invisible cracks (due to small openings/large length) can be identified***, which may make it possible to accurately determine the crack front.

**Figure 13:**
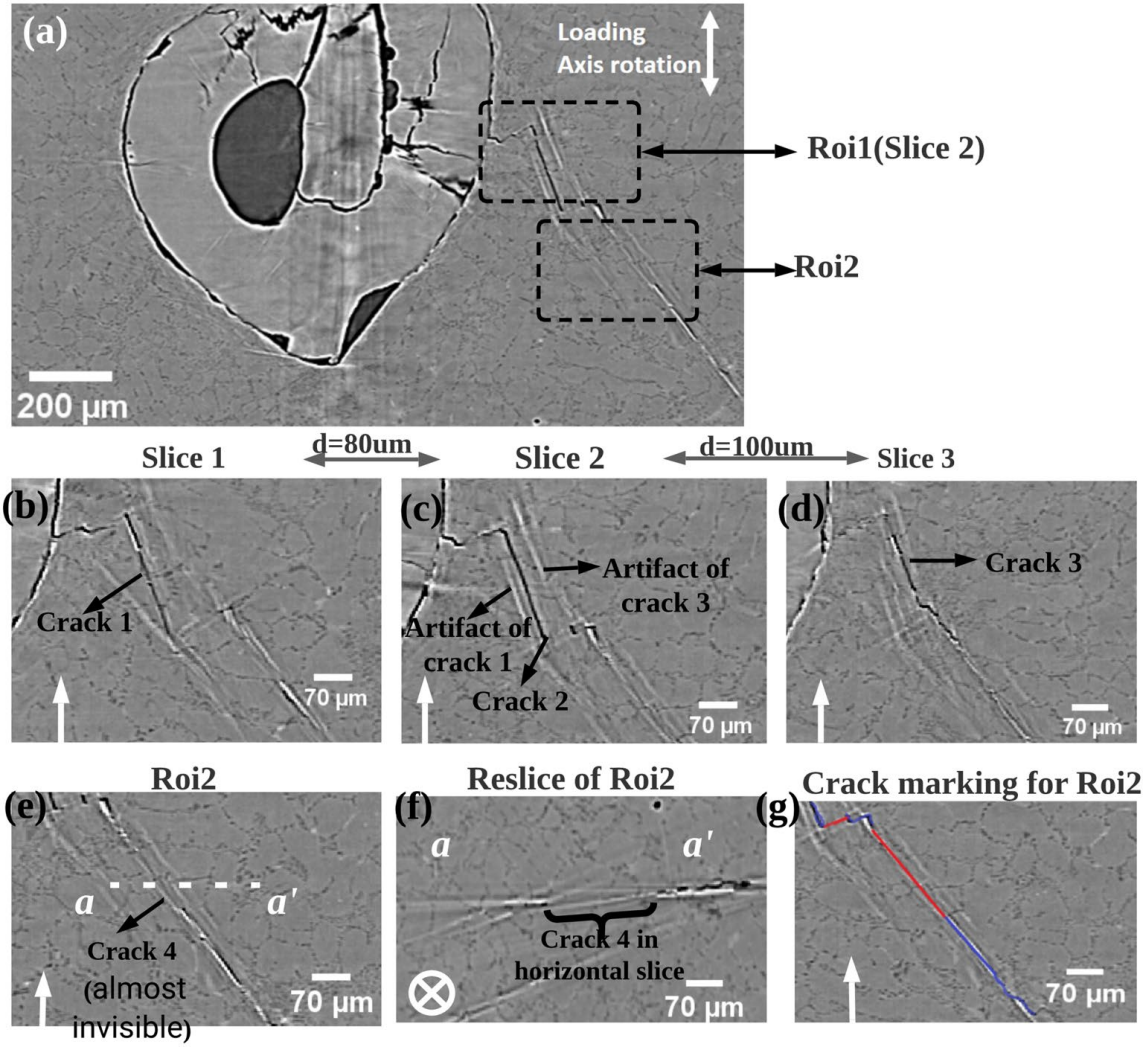
Analysis of cracks and artifacts in vertical slices of experimental scans: experimental SRCT images of cracked AlSi7 specimens which have been obtained at SLS TOMCAT beamline. The scans conditions are completely identical as Fig. 2. (**a**) A 2D experimental vertical slice, two ROIs were analyzed, in ROI1 the cracks appear dark and in ROI2 the cracks are invisible; (**b**–**d**) Enlarged view of ROI1: (**c**), the slice in front of (**c**) at a distance of 80 $\upmu$m: (**b**) and behind it at a distance of 100 $\upmu$m: (**d**); (**e**) Enlarged view of ROI2; (**f**) Horizontal re-slice of white dashed line (a–a$^{\prime }$) which shows that there is invisible crack (named crack4); (**g**) Crack marking for Roi2: markings in blue indicate a crack containing dark voxels and in red indicate an invisible crack whose presence needs to be confirmed by artifacts in horizontal slices.

By the above simulation results (Fig. 7), it can be found that the streak artifact behave differently in horizontal slices and in vertical slices. Therefore, subsequently, vertical slices of the same experimental images obtained at SOLEIL were analyzed. On Fig. 13a, the bright streak artifacts in the vertical slice do not appear at the ends of the flat crack segments but parallel to it. In ROI1 (Fig. 13c), the flat crack appears black, and then bright streak artifacts appear on both its left and right sides. With the help of simulation results (Fig. 6d,e), it is known that streak artifacts in a vertical slice indicate the presence of cracks with the identical geometry in the corresponding location of its adjacent vertical slice. Consistently, the source of the artifact on the left side of the dark crack is found in a slice ${80}\,{\upmu }m$ away from the current slice (called crack 1 in Fig. 13b) and the source of the artifact on the right side is found in the vertical slice ${100}\,{\upmu }m$ from the current slice (called crack 3 in Fig. 13d). Similar streak artifacts in vertical slices can be found in publications using phase contrast synchrotron tomography: in Al-Si cast alloys^17^; in additively manufactured Inconel^23^; in Ti alloys^31^. Our simulation results can help in distinguishing cracks from artifacts.

Another interesting case is shown in Fig. 13e. On this image, it is not clear whether a crack is present or not (indicated as crack 4). Its presence is proved by analysing an horizontal slice taken passing through the a–a$^{\prime }$ line (Fig. 13f) which shows a sub-voxel crack only signalled by the streak artifacts at its ends. The correct interpretation of the image is given by the red line in Fig. 13g. *Therefore, for internal fatigue cracks (at a certain angle e.g.,* $45{^{\circ }}$ *to the tensile direction), horizontal slices may be more convenient for complete determination of the crack tip.* To summarize, only pixels with a dark contrast can be unambiguously identified as belonging to a crack in the SRCT reconstruction image; pixels with a strong white contrast should be interpreted as streak artifacts; some cracks disappear in the image because of their small opening and large length. Phase retrieval methods could be an effective way of removing such artefacts, however they are not feasible for characterization of cracks in in-situ experiments. This is because (1) multi-distance phase retrieval methods require several distances per angle to be really efficient, which is not feasible; (2) single distance phase retrieval methods introduce some blurring of crack edges. On the other hand, the presence of these streak artifacts can assist in locating some invisible cracks. Therefore, a recently developed deep learning based method (U-net)^32^ has been used to automatically distinguish cracsks (as well as invisible cracks) from streak artifacts in SRCT images by learning observer judgment. One result is shown in “ESM Appendix”.

## Conclusions

The aim of the present paper was to better identify and characterize internal fatigue cracks and artifacts in an Al alloy in SRCT images by simulating the phase contrast contribution in the reconstructed 3D images. In the studied material, fatigue cracks grow along crystallographic planes; their surfaces form very planar facets with dimensions of the order of several 100 micrometers (cracks initiated from the surface, which has been often reported in the literature have a less planar shape for which the present analysis is less relevant). Through the simulation results, streak artifacts with shapes similar to flat cracks that cause difficulties in accurate identification of cracks were investigated. The streak artifacts always appear on both ends of the flat crack segments even when the crack is invisible. Further, in changing the size of the cracks in the simulation it has been found that the length/opening of the crack has little effect on the streak artifact and determines the gray value of the crack in the reconstructed image. The contrast in the gray value of the crack increases with the crack opening, and decreases with the crack length. Therefore, *it is very difficult to quantitatively analyze the opening degree of flat cracks through gray values*. Simulations of step-like cracks were performed and were found in good agreement with the experimental images.

The WTIE model was used to simulate the same phantom flat crack; the streak artifacts were not found on horizontal slices while they were observed when the Fresnel Propagator model was used. The central white peak of diffraction fringes observed when the latter model is used might be the reason of this difference. The gray-scale values in the cracks in the reconstructed images are consistent for the two models mentioned above. Thus, the simplified WTIE model is used for the analytical analysis of the crack gray-scale values in the reconstructed image to explain the variation of crack gray-scale values with length and opening.

Eventually, the experimental images were reanalyzed by using the results obtained from the simulation. Cracks in horizontal slices appear as dark or low contrast to the point of invisibility, and the strong white contrast parts that appear at their ends are all artifacts. The presence of those artifacts can however help to locate invisible cracks segments. The experimental step-like cracks are in good agreement with the simulation results found in the experimental horizontal slices. In vertical experimental slices, streak artifacts no longer appear at the ends of the flat crack segments; instead, they appear in the same position in the adjacent slices, and have the same shape as the crack.

## Method

### Experimental synchrotron X-ray tomography

The experimental data sets analyzed in this work were obtained with two synchrotron X-ray tomography setups at Tomcat beamline (SLS) and PSICHE beamline (SOLEIL) during in situ synchrotron fatigue tests. The scanned specimens with controlled artificial casting defect was made in AlSi7Mg0.6 cast aluminum (more information about material, specimen and fatigue testing device can be found elsewhere^10,33,34^). At SOLEIL (PSICHE), half-acquisition at a voxel size of 1.3 $\upmu$m has been performed, the energy of the filtered white beam has been set to 29 keV and the object-detector distance has been set at 15 cm. Similar scan conditions have been used at SLS (TOMCAT), but with a different voxel size (1.6 $\upmu$m) and without half acquisition. The scan conditions can be found in the Table 1.

**Table 1:** Imaging conditions used at TOMCAT and PSICHE beam-lines (FOV corresponds to the camera size expressed in pixels).

Beamline	Beam configuration	Energy	Voxel size	Distance detector-object	FOV
TOMCAT	Multilayer	30 keV	1.6 $\upmu$m	15 cm	$2560 \times 2160$
PSICHE	Filtered white beam	29 keV	1.3 $\upmu$m	15 cm	$2750 \times 2048$

### Simulation of X-ray phase contrast

**Figure 14:**
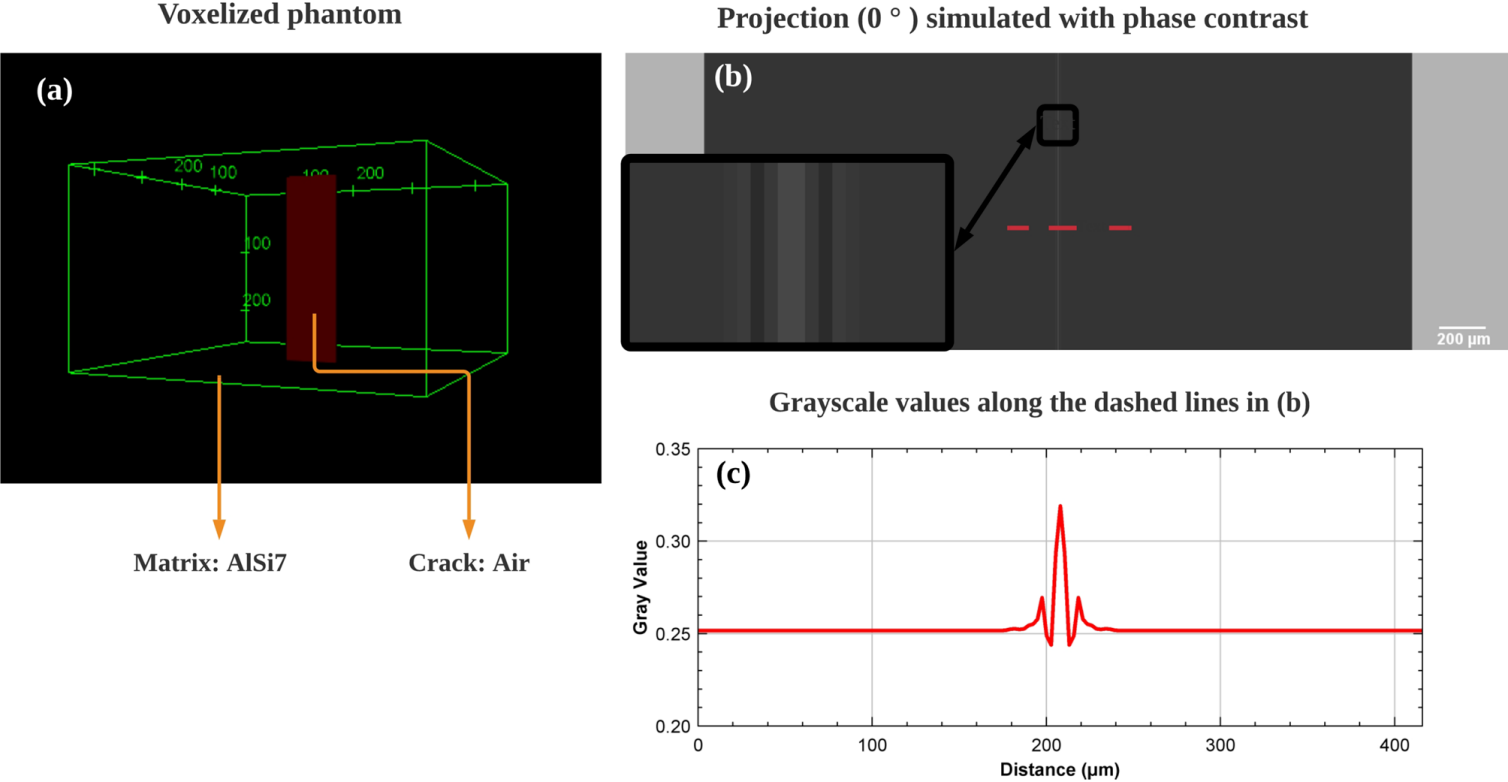
(**a**) Voxelized phantom: Rectangular block of Al-Si alloy with crack (voxel size 1 $\upmu$m). (**b**) Simulated projection(0$^\circ$) with phase contrast, the Fresnel diffraction patterns can be seen on crack interfaces. (**c**) Distribution of gray scale values along the red dashed line in the projection.

A method^20^ developed by Langer et al. was used to simulate phase contrast tomography. The first step consists in using a ray casting procedure in the voxellized object, from which the integral of the refractive index after passing through the object can be obtained. Then the Fresnel propagator is used (Eq. 10) to simulate the propagation of the X-ray in free space after passing the object from which the Fresnel diffraction patterns (as shown in Fig. 14b) are generated.

The amplitude of the wave function after free-space propagation along distance *D* (assuming parallel beam) is written as:

9 $$\begin{aligned} u_{D}(x, y)=u_{0}(x, y) * P_{D}(x, y) \end{aligned}$$

where the propagator is

10 $$\begin{aligned} P_{D}(x, y)=\frac{1}{i \lambda D} \exp \left( i \frac{\pi }{\lambda D}\left( x^{2}+y^{2}\right) \right) \end{aligned}$$

$u_{D}(x, y)$ represents the wave function received on the detector, $u_{0}(x, y)$is the wave function after the X-ray beam passes through the object, $P_{D}(x, y)$ represents the Fresnel Propagator in the time domain. In order to simplify the calculation, the convolution operation in the time domain is converted into a multiplication in the frequency domain after Fourier transform.

To implement the simulation; first, a voxelized 3D block containing a model crack (called phantom) was created as shown in Fig. 14, the rectangular shape of the scanned object is used here to avoid the effect of the jagged edges of the voxelized cylinder which is the sample real shape; secondly, different materials are defined through the gray value of the voxelized phantom (ex: voxels with gray value equal to 0 correspond to the air, voxels with gray value equal to 255 correspond to the AlSi7Mg0.6 alloy); finally the scan conditions must be defined (detector pixel size, distance between object and detector, energy of X-ray beam). The ray-casting class *GateFixedForcedDetectionActor* is used in Gate to implement the Fresnel propagator^20^. In order to deal with the partial volume effect^35^ at the interface between different materials, we had to remove the bilinear interpolation from the source code of this module. The grid size should be small enough to ensure sufficient spatial sampling. Therefore, a binning factor of 10 has been used in horizontal direction (perpendicular to rotation axis) on the detector. The simulations were implemented on a workstation with 80 CPUs and 250 GB RAM. For a simulation with $1001\times 1001\times 1001$ voxels phantom and $1200\times 1200$ detector resolution, the calculation time is about 6 mins per projection (60 h for 600 projections/180$^\circ$). Then the filtered back projection (FBP) as the standard and most popular reconstruction algorithm for parallel beam CT is used in this work.

## Supplementary Information

Supplementary Information.
