# Author Correction: Characterization of internal fatigue cracks in aluminum alloys by simulation of phase contrast tomography

**DOI:** 10.1038/s41598-023-28176-0

**Published:** 2023-01-18

**Authors:** Ce Xiao, Jean Michel Létang, Jean‑Yves Buffière

**Affiliations:** 1 Laboratoire Matériaux, Ingénierie et Sciences (MATEIS), CNRS UMR 5510, INSA‑Lyon, 69621 Villeurbanne, France; 2 INSA-Lyon, UJM-Saint Etienne, CNRS, Inserm, CREATIS UMR 5220, U1294, Université Lyon 1, University of Lyon, 69373 Lyon, France

Correction to: *Scientific Reports* 10.1038/s41598-022-09811-8, published online 08 April 2022

The original version of this Article contained an error in the Acknowledgements section.

“The Al fatigue specimen studied in this paper have been obtained through the project Gigadef (Grant number ANR16CE080039) funded by the French Agence Nationale de la Recherche. We acknowledge the provision of synchrotron radiation beamtime at SLS (Tomcat beamline) and at SOLEIL (PSICHE beamline). We thank our local contacts who helped us there: Dr Anne Bonnin at SLS and Dr Andrew King at Soleil. C. Xiao is supported by a Grant obtained in the Cleansky project IDERPLANE (Contract number CE:821315).”

now reads:

“The Al fatigue specimen studied in this paper have been obtained through the project Gigadef (Grant number ANR16CE080039) funded by the French Agence Nationale de la Recherche. We acknowledge the provision of synchrotron radiation beamtime at SLS (Tomcat beamline), at ESRF(ID11) and at SOLEIL (PSICHE beamline). We thank our local contacts who helped us there: Dr Anne Bonnin at SLS, Dr Wolfgang Ludwig at ESRF, and Dr Andrew King at Soleil. C. Xiao is supported by a Grant obtained in the Cleansky project IDERPLANE (Contract number CE:821315).”

The original Article has been corrected.

