# Supplementary material for: Characterization of internal fatigue cracks in aluminum alloys by simulation of phase contrast tomography
Source: Sci Rep. 2022 Apr 8;12:5981. doi: 10.1038/s41598-022-09811-8 (PMC8993829; doi:10.1038/s41598-022-09811-8)
Supplement: Supplementary file 1 — Supplementary Information. [file 41598_2022_9811_MOESM1_ESM.pdf]

A Appendix: Segmentation of cracks from experimental SRCT images via U-net

U-net is a deep learning based method which has been used in segmentation of SRCT images of fatigue crack[30]. With the help of the above simulation results, cracks can be clearly identified from the streak artifacts and even invisible cracks can be recognized by the streak artifacts at their ends. As shown in Figure A1a and b, cracks segments with dark contrast have been distinctly labeled (e.g. crack1); meanwhile the invisible cracks segments have been labeled by the streak artifacts at their ends (e.g. crack2). 120 slices (out of 1200 for the whole volume) were manually segmented by using the newly understood crack features. By training the U-net model using these 120 labeled images (learning rate=0.0005, batch size=100, number of epochs=100), the cracks could be successfully detected in the experimental SRCT images as can be seen in Figure A1c and d. Dark contrast cracks were undoubtedly detected, invisible cracks (e.g. crack3) were also successfully detected and no streak artifacts were misidentified as cracks.

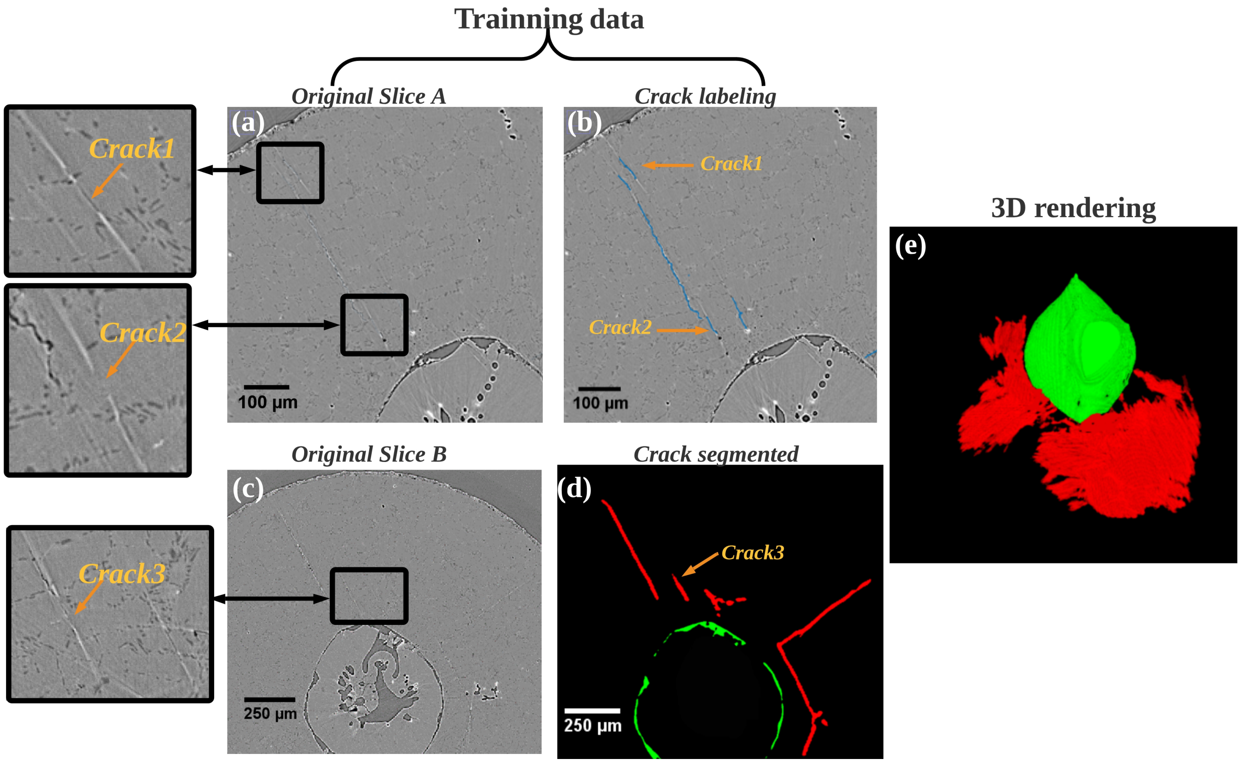

Figure A1. Segmentation results obtained with a trained U-net model (newly understood crack features have been applied during crack labeling). The segmented volume has been obtained at ESRF beamline of synchrotron ID19 (x-ray energy 29keV, sample-detctor distance 15cm, voxel size=1.3 μm). (a) 2D experimental horizontal slice (named slice A) with two zoomed ROIs, crack1 appears as light gray with streak artifacts at ends and crack2 is INVISIBLE with streak artifacts; (b) Crack labeling of Slice A; (c) Another 2D experimental horizontal slice (named slice B), crack3 is INVISIBLE with streak artifacts; (d) Segmentation results of Slice B, crack3 has been successfully detected; (e) 3D rendering of cracks.
